# Integrating Metabolomics and Genomics to Uncover Antimicrobial Compounds in *Lactiplantibacillus plantarum* UTNGt2, a Cacao-Originating Probiotic from Ecuador

**DOI:** 10.3390/antibiotics14020123

**Published:** 2025-01-24

**Authors:** Diana Molina, Evelyn Angamarca, George Cătălin Marinescu, Roua Gabriela Popescu, Gabriela N. Tenea

**Affiliations:** 1 Biofood and Nutraceutics Research and Development Group, Faculty of Engineering in Agricultural and Environmental Sciences, Universidad Técnica del Norte, Ibarra 100150, Ecuador; dmmolinap@utn.edu.ec (D.M.); elangamarcac@utn.edu.ec (E.A.); 2 Asociația Independent Research, 58 Timișului, Sector 1, 012416 Bucharest, Romania; catalin.marinescu@independent-research.ro (G.C.M.); roua.popescu@independent-research.ro (R.G.P.); 3 Blue Screen SRL, 58 Timișului, Sector 1, 012416 Bucharest, Romania

**Keywords:** probiotics, metabolites, antimicrobials, lanthipeptides, LC-MS/MS untargeted metabolomics

## Abstract

**Background/Objectives**: Lactic acid bacteria (LAB) produce several diverse metabolites during fermentation that play key roles in enhancing health and food quality. These metabolites include peptides, organic acids, exopolysaccharides, and antimicrobial compounds, which contribute to gut health, immune system modulation, and pathogen inhibition. This study analyzed the intracellular (Met-Int) and extracellular metabolites (Met-Ext-CFS; cell-free supernatant) of *Lactiplantibacillus plantarum* UTNGt2, a probiotic strain isolated from *Theobroma grandiflorum*. **Methods**: The assessment was performed using capillary LC-MS/MS metabolomics with a SWATH-based data-independent acquisition approach to identify molecules associated with antimicrobial activity. **Results**: The integration of metabolomic data with whole-genome annotation enabled the identification of several key metabolites, including amino acids, nucleotides, organic acids, oligopeptides, terpenes, and flavonoids, many of which were associated with the antimicrobial activity of UTNGt2. Pathway analysis reveals critical processes such as secondary metabolite biosynthesis, nucleotide and galactose metabolism, and cofactor biosynthesis. By integrating RiPP (ribosomally synthesized and post-translationally modified peptide) cluster gene predictions with LC-MS data, this study validates the production of specific RiPPs and uncovers novel bioactive compounds encoded within the UTNGt2 genome. The oligopeptide val-leu-pro-val-pro-gln found in both Met-Int (ESI+) and Met-Ext-CFS (ESI+) may contribute to the strain’s antimicrobial strength. It could also enhance probiotic and fermentation-related functions. **Conclusions**: While genome-based predictions highlight the strain’s biosynthetic potential, the actual metabolite profile is influenced by factors like transcriptional regulation, post-transcriptional and post-translational modifications, and environmental conditions. These findings emphasize the value of multi-omics approaches in providing a holistic understanding of metabolite production and its role in antimicrobial activity.

## 1. Introduction

Food contamination by pathogens, a leading cause of foodborne illnesses, remains a significant challenge for the food industry [1]. A practical solution is to prioritize safe foods containing fewer chemical additives and more natural ingredients, which preserve organoleptic qualities and ensure consumer safety [2]. While additive-free foods are generally preferred, when unavailable, consumers tend to favor those with natural additives over synthetic ones [3]. Thus, LAB, an abundant source of bioactive compounds or metabolic products, are considered a natural alternative to preserve food quality and safety [4]. LAB can degrade food macromolecules, including indigestible polysaccharides and undesirable flavor compounds while producing various metabolites, such as vitamins, amines, bacteriocins, short-chain fatty acids, and exopolysaccharides [5]. These metabolic traits have broadened their applications in the food industry, where they are utilized to enhance nutritional value, reduce toxic substances, extend shelf life, and improve the flavor of fermented foods [4]. In addition, LAB specifically compete with pathogens for nutrients, bind to receptors, and generate antimicrobial compounds such as bacteriocins [1]. These bacteriocins fit into four classes: Class I (modified and lanthionine; antibiotics and nisin), Class II (unmodified and linear heat-stable non-lanthionine; <10 kDa), Class III (large heat–labile peptides; >30 kDa), and Class IV (small and circular peptides; <10 kDa) [6]. Bacteriocins that differ in their mode of action, molecular weight, genetic origin, and biochemical characteristics are secreted by different strains [7]. Their ability to disrupt membrane vesicles by acting on the bacterial cytoplasmic membrane may be advantageous when using LAB to target a particular sensitive pathogen [7]. The emerging evidence suggests that the health benefits of LAB may stem not only from the bacteria themselves but also from their bacteriocins and other metabolites and components, collectively known as postbiotics [8]. Postbiotics exhibit various biological activities, including antimicrobial, antioxidant, anti-inflammatory, anti-proliferative, and immunomodulatory effects, with significant potential for disease treatment [9]. For food and feed manufacturers, these metabolites can inhibit pathogen growth and, through interactions with the host and other microbes in the same niche, may play a crucial role in combating infections [10].

Recently, we demonstrated that CFS derived from several native LAB species can inhibit foodborne pathogens [11]. This effect was attributed to the direct interaction of antimicrobial molecules with the pathogens that impair their growth and colonization. In addition, these metabolites disrupted the target cell integrity by releasing aromatic molecules from the cytoplasm, as well as inducing structural membrane damage and cell wall holes of the target strain [12]. Particularly, the whole-genome annotation of the *Lactiplantibacillus plantarum* strain UTNGt2, isolated from white cacao fruits, revealed specific bacteriocin cluster genes that may contribute to its antimicrobial activity [11]. The strain produces sactipeptide-class (Class IIc) bacteriocins, plantaricin E, and several lanthipeptides, along with non-ribosomal peptide synthase (NRPS) clusters [11]. This genomic diversity suggests an adaptation to its niche-specific lineage and environment. Additionally, using targeted genome mining tools, we identified a rich arsenal of antimicrobial molecules, such as lanthipeptides, while shedding light on their natural biosynthesis pathways. However, due to gaps in the existing databases and the lack of functional validation, genome annotation of a strain isolated from a specific niche may overlook novel or poorly characterized antimicrobial molecules, or antimicrobial gene clusters may be present but remain inactive under standard laboratory conditions, making their functional roles difficult to predict from genome data alone [13]. Additionally, while genome annotation provides genetic information, it cannot determine the exact chemical structures or bioactivity of the antimicrobial compounds without complementary metabolomic analysis [14]. Therefore, to improve our knowledge of the antimicrobial strength of the *L. plantarum* UTNGt2 strain, especially to identify the metabolites with potential inhibitory action, the current study further investigates the composition of both internal (Met-Int) and external (Met-Ext-CFS) metabolites using a capillary LC-MS/MS metabolomics method with a SWATH-based data-independent acquisition strategy for simultaneous targeted and untargeted metabolites. In addition, these data were corroborated with the metabolite analysis retrieved from UTNGt2 whole-genome annotation.

## 2. Results and Discussion

### 2.1. Internal (Intracellular) Metabolite Identification

**Figure 1:**
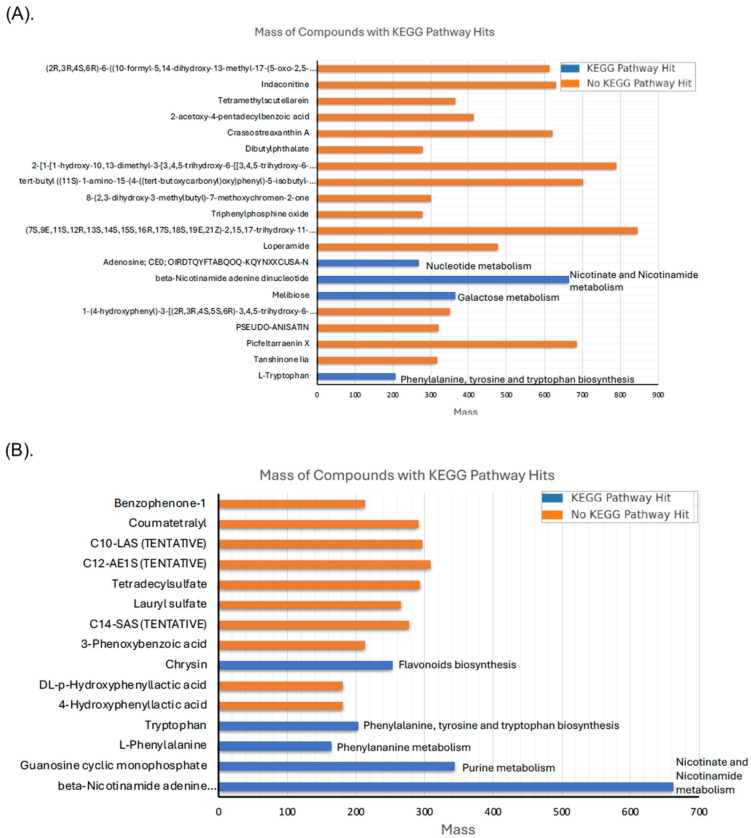
Mass of intracellular compounds with KEGG pathway hits. (**A**) Met-Int-ESI (+) and (**B**) Met-Int-ESI (−).

A total of 3237 precursor molecules were extracted from the peak chromatograms in positive and negative ion modes, respectively. After filtering (0.01 Da MS1 tolerance and 0.025 Da MS2 tolerance), 20 Met-Int (ESI+) metabolites and 15 Met-Int (ESI−) were identified by mass-to-charge matching (*m*/*z*), and their identity was confirmed using MS/MS matched data (tandem mass spectrometry). Table 1 and Table 2 show the compound list, class, formula, mass, and hits with several databases. Detailed information on these metabolites, including their ontology, mass-to-charge ratio (*m*/*z*), exact mass, molecular formula, mass error, and precursor type, is provided in Tables S1 and S2. In complex biological samples, multiple metabolites often co-elute, resulting in overlapping precursor ions and mixed product ion spectra (chimeric spectra). Deconvolution separates these overlapping signals, enabling the accurate identification of individual metabolites [15]. Figure S1 depicts a comparison of the deconvoluted spectra with reference spectra for some compounds detected in the Met-Int ESI+ sample, which may be related to the antimicrobial capacity. For example, the close alignment peaks at 301.14 and 413.26 *m*/*z* of 2-acetoxy-4-pentadecylbenzoic acid indicate the sample likely contains the compound or a close structural analog of the reference, and the unmatched peaks indicate structural differences (e.g., isomers or additional functional groups). The results demonstrate that the Met-Int (ESI+) extract contains several functional metabolites of 16 classes, such as amino acids, nucleotides, terpenes, coumarins, oligopeptides, benzoic acid esters, flavonoids, benzaldehydes, benzene and substitute derivates, fatty acyl glycosides of mono- and disaccharides, macrolactams, with anolide glycosides and derivatives, aconitane-type diterpenoid alkaloids, and cardenolide glycosides and derivatives. Some organic compounds such as benzoic acid, 8-(2,3-dihydroxy-3-methylbutyl)-7-methoxychromen-2-one, tert-butyl ((11S)-1-amino-15-(4-((tert-butoxycarbonyl)oxy)phenyl)-5-isobutyl-11-methyl-1,4,7,10,13-pentaoxo-3,6,9,12-tetraazapentadecan-14-yl)carbamate, tetramethylscutellarein, and C_45_H_62_N_2_O_12_ showed two or three peaks, suggesting the presence of isomers in the sample. Benzoic acid has been previously detected in lactic acid bacteria and is mainly used as a food preservative [16]. Apart from being utilized to prevent microbial spoilage in foods, it can also develop naturally in foods under certain conditions [17]. Compound 8-(2,3-dihydroxy-3-methylbutyl)-7-methoxychromen-2-one belongs to the coumarin class of compounds, which are detected mainly in plant extracts [18]. In addition, tetramethylscutellarein, a 7-O-methylated flavonoid, is a hydrophobic and water-insoluble lipid molecule [19]. Pathway analysis of the metabolome highlights several matched pathways, determined by significant *p*-values in the enrichment analysis and impact values in the topology analysis. These pathways include biosynthesis of secondary metabolites, nucleotide metabolism, galactose metabolism, and cofactor biosynthesis (Figure 1A). Likewise, the Met-Int (ESI−) extract contains several functional metabolites of 10 classes, including organic acids, nucleotides, amino acids, flavones, sulfuric acids monoesters, etc. (Table 2). Among them, lauryl acid, 3-phenoxybenzoic acid, and 4-hydroxyphenyllactic acid showed inhibitory action against fungi and bacteria [20]. Interestingly, 4-hydroxyphenyllactic acid (4-HO-PLA), an antifungal compound, was previously studied in *Lactobacillus* sp. SK007 growth [21]. A recent study using LC-MS metabolomics identified seven metabolites from the *L. plantarum* DSMZ 13890 strain, including 4-HO-PLA that showed antifungal capacity [22]. In addition, an interesting compound chrysin, a natural polyphenol, was detected. Normally, it is found in honey, propolis, and fruits and has a wide range of biological activities, including the prevention of oxidative stress, inflammation, neurodegeneration, and carcinogenesis [23]. In our previous study, the inoculation of UTNGt2 cells into a cacao-based beverage significantly enhanced its antioxidant activity and polyphenol content. This improvement is likely attributed to the bioactive compounds released during the fermentation process [24]. Metabolome pathway analysis revealed matched pathways, such as nicotinate and nicotinamide metabolism, purine metabolism, phenylalanine metabolism, and phenylalanine, tyrosine, and tryptophan biosynthesis, as well as glycine, serine, and threonine metabolism (Figure 1B). The uncommon, detected metabolites such as terpene or alkaloids found in bacteria could indicate the acquisition of biosynthetic pathways through horizontal gene transfer (HGT) [25]. HGT allows bacteria to incorporate genes from other organisms (i.e., fungi, plants, or other bacteria), leading to the production of metabolites not typically associated with bacteria. Considering the origin of the UTNGt2 is from cacao fruit, the detection of these compounds may suggest bacterial adaptation, survival strategies, or symbiotic interactions where such genes were acquired from the cacao plant.

### 2.2. External (Extracellular) Metabolite Identification

**Figure 2:**
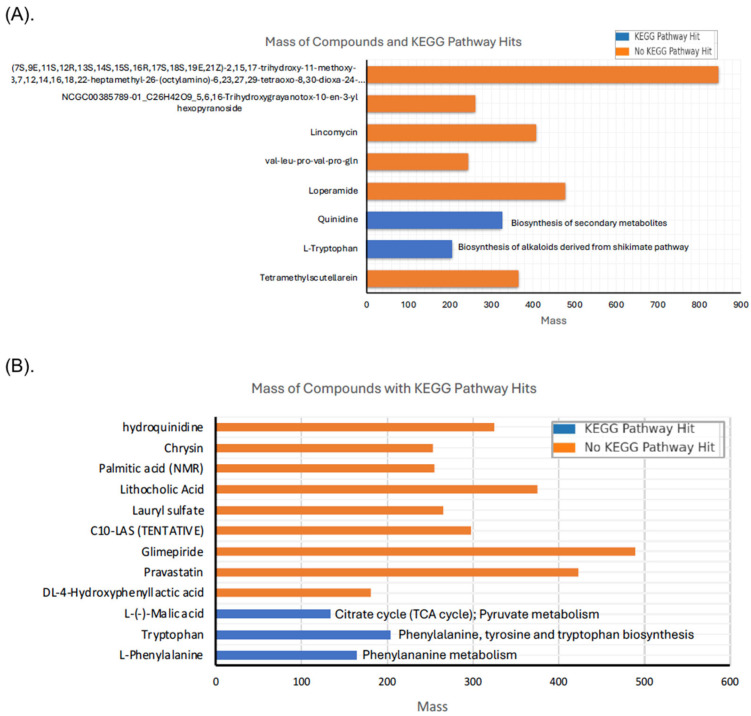
Mass of Extracellular compounds with KEGG pathway hits. (**A**) Met-Ext-CFS-ESI (+) and (**B**) Met-Ext-CFS-ESI (−).

A total of 243 precursor molecules were extracted from the peak chromatograms in positive ion mode and 1312 in negative ion mode. Among these, 8 Met-Ext-CFS (ESI+) and 12 Met-Ext-CFS (ESI−) compounds were identified through MS/MS matching, representing various classes, such as alkaloids, flavonoids, amino acids, proline derivatives, macrolactams, O-glycosyl compounds, and benzene derivatives. Figure S2 presents a comparison between the deconvoluted spectra and reference spectra for selected compounds detected in the Met-Ext-CFS (ESI+) sample. Table 3 provides a detailed list of the identified compounds, including their class, formula, mass, and database matches. Similarly, a recent LC-MS analysis of *Lactobacillus paracasei* MRS-4 CFS identified 97 compounds, including organic acids, polypeptides, and amino acids, with 43 organic acids exhibiting antibacterial activity against *Alicyclobacillus acidoterrestris* [20]. Additionally, KEGG pathway analysis linked two compounds to the biosynthesis of secondary metabolites and alkaloids derived from the shikimate pathway (Figure 2A). Likewise, the compounds detected in the negative mode (ESI-) indicated the presence of several amino acids, such as tryptophan and phenylalanine, organic acids, malate, and DL-4-hydroxyphenyllactic acid, as well as fatty acids, monoesters, monohydroxy bile acids, alcohols and derivatives, and flavonoids (Table 4). Details of the detected compounds and orthologs are shown in Table S4. Fatty acids and their derivatives serve as natural, effective antimicrobial agents in food [26]. DL-4-hydroxyphenyllactic acid is a derivative of lactic acid, where one of the methyl hydrogens is replaced by a 4-hydroxyphenyl group, and it is produced by lactobacilli [21]. Its production as a novel antifungal compound was investigated during the growth of *Lactobacillus* sp. SK007 [21]. The detection of lithocholic acid suggests a link between UTNGt2 metabolism and bile acid transformation, highlighting potential adaptations to a bile-rich environment like the gut [27]. It also raises interesting possibilities regarding microbial competition, host interactions [28], and the unique metabolic capabilities of the UTNGt2 strain. Further investigations could focus on identifying the metabolic pathways involved in lithocholic acid production, exploring bile acid tolerance mechanisms in *Lactobacillus*, and examining its bioactivity and ecological or functional role in the microbiome. The pathways analysis linked several compounds with phenylalanine metabolism; phenylalanine, tyrosine, and tryptophan biosynthesis; and glycine, serine, and threonine metabolism, as well as pyruvate metabolism (Figure 2B). This study underscores the metabolic versatility of *UTNGt2,* with the detection of compounds relevant to antimicrobial activity, bile acid transformation, and amino acid metabolism. These findings reveal promising avenues for understanding bacterial adaptations, functional metabolite production, and their potential biotechnological applications.

### 2.3. RiPP Prediction from UTNGt2 Genome and Metabolome

**Figure 3:**
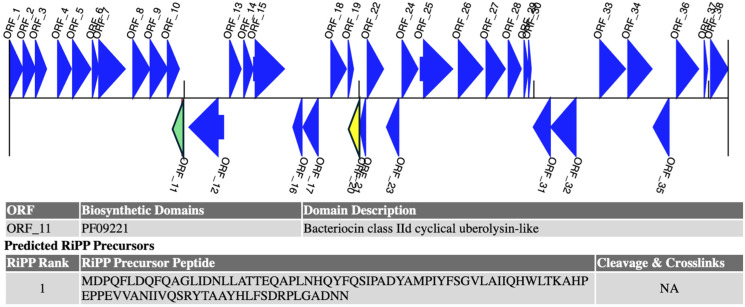
Biosynthetic gene cluster and predicted RiPP domain in the UTNGt2 genome. Green arrow: biosynthetic; Yellow arrow: small ORFs; blue arrow: other small ORFs. ORF: open reading frame.

Microorganisms can produce a vast array of natural products, which are synthesized by specific biosynthetic gene clusters [29]. They are classified into two groups: ribosomally synthesized and post-translationally modified peptides (RiPPs) and non-ribosomal peptides (NRPs) [30]. RiPPs, such as lanthipeptides and lasso peptides, undergo ribosomal synthesis with complex modifications, while NRPs are synthesized independently of the ribosome. RiPPs are explored as novel therapeutics (e.g., antibiotics, antivirals, and anticancer agents), eco-friendly biopesticides, and natural food preservatives, like nisin, making them valuable across multiple sectors [31]. From the genome annotation, one RiPP cluster in contig 8 (starts at 20529 and ends at 61647) of the head-to-tail cyclized RiPP class with the biosynthetic domain of bacteriocin Class IId cyclical uberolysin-like (ORF_11) was predicted in the UTNGt2 genome (Figure 3). These bacteriocins are membrane-interacting peptides produced by several Firmicutes [32]. Although with low similarity, several RiPP-like compounds were predicted by antiSMASH in the contig 22 of UTNGt2 genome [11]. These metabolites, such as coagulin, microcin M, biceurecin, pallidocin, sublacin 168, etc., showed cluster similarities with other *L. plantarum* species from the database.

Moreover, the chemical structure search in the RiPP database of the 20 Met-Int (ESI+) metabolites obtained by LC-MS identified similarities with several RiPPs categorized into groups such as thiopeptides, lassopeptides, lanthipeptides, and cyanobactins (Table S5). Based on their molecular structures, L-tryptophan and tert-butyl ((11S)-1-amino-15-(4-((tert-butoxycarbonyl)oxy)phenyl)-5-isobutyl-11-methyl-1,4,7,10,13-pentaoxo-3,6,9,12-tetraazapentadecan-14-yl)carbamate showed the highest similarity (0.68) to lassomycin, a lassopeptide derived from the bacterium *Lentzea kentuckyensis*, and hymenamide K, a cyanobactin from the marine sponge *Hymeniacidon* sp., respectively. Previous studies showed that lassomycin, a highly basic, ribosomally encoded cyclic peptide, exhibits potent bactericidal activity against both growing and dormant mycobacteria, including drug-resistant forms of *M. tuberculosis* [33]. Additionally, tetramethylscutellarein, loperamide, indaconitine, and crassostreaxanthin A exhibited moderate similarity to other peptides: the thiopeptide JBIR-83 from *Streptomyces* sp. RI19, the lanthipeptide hominicin from *Staphylococcus hominis* MBBL, the thiopeptide siomycin C from *Streptomyces sioyaensis* ATCC 13989, and the thiopeptide thiocillin from *Bacillus cereus* ATCC 14579. This indicates shared structural features, with varying degrees of relatedness. Likewise, 2-acetoxy-4-pentadecylbenzoic acid. Pseudo-anisatin, tanshinone Iia, and dibutylphthalate exhibited moderate similarity to various cyanobactins (Table S5). Cyanobactins are small cyclic peptides synthesized by a wide variety of cyanobacteria found in symbiotic relationships and terrestrial, marine, or freshwater environments. These compounds exhibit activities such as antimalarial, antitumor, and multidrug resistance reversal, making them promising candidates for pharmaceutical development [34] Additionally, analysis of the chemical structure similarity of the eight metabolites identified in Met-Ext-CFS (ESI+) in the RiPP database reveals that the oligopeptide val-leu-pro-val-pro-gln exhibited an identical structural match to zucinodin, a lassopeptide identified in *Phenylobacterium zucineum* HLK1 (Table S6). Lassopeptides are peptide-based natural products that represent a valuable source of medically significant compounds [35]. Their biosynthesis in nature is either directed by the ribosomal translation of the genetic code or occurs through ribosome-independent mechanisms. However, we hypothesize that the oligopeptide val-leu-pro-val-pro-gln could play a role in the antimicrobial action of the UTNGt2 CFS extract. When *Salmonella* cells were treated with the UTNGt2 peptide extract at a final concentration of 1 X MIC, spheroplast formation was observed. The cells exhibited a change in shape, with both the inner and outer membranes remaining intact, but the peptidoglycan layer was lost. Increasing the peptide concentration to 2 X MIC resulted in the formation of spheroplasts along with “ghost cells”, indicating that the target bacteria were nearly or completely devoid of cytoplasm [12]. The disruption of the cell membrane observed upon treatment with UTNGt2 peptide–protein extracts suggests that this oligopeptide could be a promising candidate for antibiotic research. Interestingly, lincomycin, a well-known antibiotic found in *Streptomyces*, exhibited moderate similarity (0.45) with sublancin 168, a glycocin from *Bacillus subtilis* strain 168. This RiPP was also predicted by antiSMASH, highlighting the strong antimicrobial potential of this strain, as multiple compounds contribute to the overall inhibitory activity [11]. In addition, loperamide and tetramethylscutellarein were identified in both Met-Int (ESI+) and Met-Ext-CFS (ESI+), which suggests that the bacteria are actively exporting certain compounds into the extracellular environment, and it is likely these compounds are linked to the antimicrobial activity, though further studies are needed to validate this statement. 

The mismatch between metabolites predicted by LC-MS and those inferred from genome analysis can be attributed to the complexities of post-transcriptional and post-translational modifications, regulatory mechanisms, and environmental factors that influence metabolite expression [36]. While LC-MS detects the actual metabolites present in a sample relying on factors such as gene expression, post-translational modifications, and metabolite stability, genome analysis predicts RiPPs based on biosynthetic gene clusters, which provide the genetic blueprint for peptide synthesis [37]. However, not all predicted peptides are necessarily produced or detectable under the specific experimental conditions used. For instance, while biosynthetic genes may be transcribed, the corresponding RNA might undergo degradation, editing, or alternative splicing, which can affect the production of the predicted metabolites [37]. Such processes may alter or suppress the synthesis of specific compounds anticipated from a genomic analysis [37]. Additionally, RiPPs and other secondary metabolites undergo significant post-translational modifications, such as heterocyclization, oxidation, methylation, or glycosylation [38]. Variations or failures in these modifications can result in final products that differ from genomic predictions [39]. Furthermore, some metabolites may be synthesized in quantities below the detection limits of LC-MS, contributing to the observed discrepancies between genomic predictions and metabolomic data [40].

## 3. Materials and Methods

### 3.1. Bacterial Culture and Metabolite Extraction

The *Lactiplantibacillus plantarum* strain UTNGt2 (GenBank accession No. KY041688.1) was previously isolated from white cacao fruits [11]. The strain was maintained as frozen stock cultures in MRS broth (Difco, Detroit, MI, USA). For the extraction of both internal (Met-Int) and external metabolites (Met-Ext-CFS), an overnight culture of UTNGt2 was obtained upon cultivation in MRS broth at 37 °C for 24 h. Further, the culture was centrifuged at 13,000× *g* for 30 min at 4 °C to separate the CFS from the cells [11]. The CFS was filtered through a 0.22 µm syringe filter (#STF020025H, Chemlab Group, Washington, DC, USA) and stored at 4 °C for subsequent analysis. The Met-Int was extracted by treating the cells with a methanol/water mixture (HPLC-MS grade, 4:1 *v*/*v*). The extraction process included three freeze–thaw cycles using liquid nitrogen, followed by incubation at 35 °C for 5 min, brief vortexing, and sonication at 20 kHz for 2 min on ice to obtain the cell lysate. The mixture was then centrifuged at 13,000× *g* for 10 min at 4 °C to collect the supernatant (Met-Int). Finally, both Met-Ext-CFS and Met-Int were lyophilized before use.

### 3.2. Capillary LC-MS/MS

The samples were centrifuged at 17,000× *g*, and the supernatant was transferred to vials for LC-MS/MS analysis using an AB SCIEX TRIPLE TOF 5600+ mass spectrometer (Sciex, Concord, ON, Canada). Chromatographic separation was performed on a NanoLC 425 system (Eksigent, Dublin, CA, USA), with an Eksigent 5C18-CL-120 analytical column (300 μm ID and 150 mm length) connected to an AB Sciex DuoSpray ion source. The mass spectrometer was calibrated automatically for every three samples, and the calibration pass criteria were mass accuracy better than 4 ppm and ensuring accuracy for the next five hours of better than 20 ppm, which corresponds to 0.01 Da at 500 *m*/*z*. A 5 μL sample was injected and subjected to a gradient elution of 5% to 80% acetonitrile containing 0.1% formic acid over 90 min at a flow rate of 5 μL/min, with the column temperature maintained at 55 °C. Electrospray ionization was employed in both positive and negative modes. For the positive ion mode, the source parameters were GS1: 7, GS2: 0, CUR: 20, TEM: 0, and ISVF: 5500 V, while for the negative ion mode, the settings were GS1: 20, GS2: 0, CUR: 25, TEM: 0, and ISVF: 4500 V. The TRIPLE TOF 5600+ was operated in DIA SWATH-MS mode with 60 variable windows, following a previously established protocol [41].

### 3.3. SWATH Data Acquisition

MS1 survey scans covered a range between 100 and 1250 *m*/*z*, and MS2 spectra were acquired in a high-sensitivity mode between 100 and 2000 *m*/*z*, with accumulation times set to 150 ms for MS1 and 30 ms for MS2, producing a 2 s cycle. Collision energy (CE) was automatically optimized by Analyst TF 1.8.1 software for each SWATH window, with the CE spread (CES) set to 15 V.

### 3.4. Data Processing and Identification

Untargeted metabolite identification from the SWATH data was performed using MS-DIAL version 5.3.240719, with metabolite annotation based on all relevant databases provided in the MSP spectral kit (https://systemsomicslab.github.io/compms/msdial/main.html#MSP, accessed on 26 October 2024). For mass accuracy, we applied a 0.01 Da tolerance for MS1 and a 0.025 Da tolerance for MS2.

### 3.5. Prediction of Ribosomally Synthesized and Post-Translationally Modified Peptides (RiPPs) from Genome and Metabolome Analyses

The RiPPMiner-Genome tool was used to identify biosynthetic gene clusters (BGCs) for RiPPs (ribosomally synthesized and post-translationally modified peptides) from genomic sequences [42]. Moreover, the chemical structure in the SMILE format retrieved from LC–MS data was analyzed with a RiPP miner web interface to detect the similarity with ribosomally synthesized and post-translationally modified peptides (RiPPs) from other organisms [42]. This analysis is based on the detection of 10 similar structures according to the Tanimoto score [43]. It is based on comparing molecular fingerprints, which are binary representations of molecular characteristics, like the presence or absence of specific functional groups or substructures. The Tanimoto score categorizes molecular similarity into five levels, ranging from very low (0.0–0.2: few common features) to very high or identical (0.8–1.0: highly similar or identical structures).

### 3.6. Prediction of Metabolite Pathways

The metabolites identified by LC–MS were linked to different pathways through Metabolomics Pathway Analysis version 6.0 (MSEA) (http://www.metaboanalyst.ca/, accessed on 15 November 2024) [44]. The uploaded compounds list was converted by a built-in tool into common names, synonyms, and identifiers used in HMDB ID, PubChem, and KEGG databases. The Kyoto Encyclopedia of Genes and Genomes (KEGG) database (http://www.kegg.jp/, accessed on 15 November 2024) was utilized to map these metabolites and determine their roles in metabolic pathways [45].

## 4. Conclusions

By combining LC-MS analysis with RiPP cluster gene predictions, we correlated the detected metabolites with genomic annotations, successfully validating specific RiPPs and identifying novel bioactive compounds. Notable metabolites, such as 4-hydroxyphenyllactic acid, benzoic acid, lauric acid, and the oligopeptide val-leu-pro-val-pro-gln, are likely associated with potent antimicrobial activity. However, further assays are required to confirm that these fractions are directly responsible for the observed antimicrobial effects. Genome analysis revealed RiPP biosynthetic clusters, including lassopeptides, thiotepides, and lanthipeptides, known for their antimicrobial, antifungal, and antitumor properties. Pathway enrichment analysis further identified pathways involved in secondary metabolite production, nucleotide synthesis, and nicotinamide biosynthesis. Although genomic predictions highlight the biosynthetic potential, the metabolite profiles are ultimately shaped by transcriptional, post-transcriptional, and environmental factors. Importantly, this is the first study to analyze the metabolites of *L. plantarum* UTNGt2 by integrating genomics and metabolomics, providing a comprehensive understanding of its biosynthetic capabilities. These findings not only advance the understanding of *L. plantarum* UTNGt2 but also pave the way for applications in developing antimicrobial agents, functional foods, and other biotechnological innovations.

## Figures and Tables

**Table 1:** List of internal metabolites (Met-Int ESI+), classification, chemical formulas, molecular masses, and their corresponding matches (or lack thereof) in various biochemical databases, including HMDB (Human Metabolome Database), PubChem, and KEGG.

Compound	Class	Formula	Mass	HMDB	PubChem	KEGG
L-Tryptophan	Amino acids	C_11_H_12_N_2_O_2_	205.10	HMDB0000929	6305	C00078
Tanshinone Iia	Terpene	C_19_H_18_O_3_	317.11	-	-	-
Picfeltarraenin X		C_36_H5_4_O_11_	685.35	-	-	-
Pseudo-anisatin		C_15_H_22_O_6_	321.13	-	-	-
1-(4-hydroxyphenyl)-3-[(2R,3R,4S,5S,6R)-3,4,5-trihydroxy-6-(hydroxymethyl)oxan-2-yl]oxypropan-1-one	Fatty acyl glycosides of mono- and disaccharides	C_15_H_20_O_8_	351.11	-	-	-
Melibiose		C_12_H_22_O_11_	365.11	HMDB0000048	6602503	C05402
Beta-nicotinamide adenine dinucleotide	Nucleotide	C_21_H_27_N_7_O_14_P_2_	664.12	HMDB0000902	5892	C00003
Adenosine; CE0; OIRDTQYFTABQOQ-KQYNXXCUSA-N		C_10_H_13_N_5_O_4_	268.10	HMDB0000050	60961	C00212
Loperamide	Benzene and substituted derivatives	C_29_H_33_ClN_2_O_2_	477.23	HMDB0004999	3955	C07080
(7S,9E,11S,12R,13S,14S,15S,16R,17S,18S,19E,21Z)-2,15,17-trihydroxy-11-methoxy-3,7,12,14,16,18,22-heptamethyl-26-(octylamino)-6,23,27,29-tetraoxo-8,30-dioxa-24-azatetracyclo [23.3.1.1^4,7^.0^5,28^]triaconta-1,3,5(28),9,19,21,25-heptaen-13-yl acetate	Macrolactams	C_45_H_62_N_2_O_12_	845.42	-	-	-
Triphenylphosphine oxide	Benzaldehydes	C_18_H_15_OP	279.09	-	-	-
8-(2,3-dihydroxy-3-methylbutyl)-7-methoxychromen-2-one	Coumarins	C_15_H_18_O_5_	301.10	-	-	-
Tert-butyl ((11S)-1-amino-15-(4-((tert-butoxycarbonyl)oxy)phenyl)-5-isobutyl-11-methyl-1,4,7,10,13-pentaoxo-3,6,9,12-tetraazapentadecan-14-yl)carbamate	Oligopeptide	C_32_H_50_N_6_O_10_	701.35	-	-	-
2-[1-[1-hydroxy-10,13-dimethyl-3-[3,4,5-trihydroxy-6-[[3,4,5-trihydroxy-6-(hydroxymethyl)oxan-2-yl]oxymethyl]oxan-2-yl]oxy-2,3,4,7,8,9,11,12,14,15,16,17-dodecahydro-1H-cyclopenta[a]phenanthren-17-yl]ethyl]-4,5-dimethyl-2,3-dihydropyran-6-one	Withanolide glycosides and derivatives	C_40_H_62_O_14_	789.40	-	-	-
Dibutylphthalate	Benzoic acid esters	C_16_H_22_O_4_	279.16	-	-	-
Crassostreaxanthin A	Diterpene glycosides	C_40_H_54_O_4_	621.39	-	-	-
2-acetoxy-4-pentadecylbenzoic acid	Benzoic acids	C_24_H_38_O_4_	413.27	-	-	-
Tetramethylscutellarein	Flavonoids	C_19_H_18_O_6_	365.10	HMDB0030575	96118	C14472
Indaconitine	Aconitine-type diterpenoid alkaloids	C_34_H_47_NO_10_	630.32	-	-	-
(2R,3R,4S,6R)-6-((10-formyl-5,14-dihydroxy-13-methyl-17-(5-oxo-2,5-dihydrofuran-3-yl)hexadecahydro-1H-cyclopenta[a]phenanthren-3-yl)oxy)-4-methoxy-2-methyltetrahydro-2H-pyran-3-yl acetate	Cardenolide glycosides and derivatives	C_32_H_46_O_10_	613.29	-	-	-

**Table 2:** List of internal metabolites (Met-Int ESI−), classification, chemical formulas, molecular masses, and their corresponding matches (or lack thereof) in various biochemical databases, including HMDB (Human Metabolome Database), PubChem, and KEGG.

Compound	Class	Formula	Mass	HMDB	PubChem	KEGG
Beta-nicotinamide adenine dinucleotide	Nucleotides	C_21_H_27_N_7_O_14_P_2_	662.10	HMDB0000902	5892	C00003
Guanosine cyclic monophosphate		C_10_H_12_N_5_O_7_P	344.04	HMDB0001314	24316	C00942
L-Phenylalanine	Amino acids	C_9_H_11_NO_2_	164.07	HMDB0000159	6140	C00079
Tryptophan		C_11_H_12_N_2_O_2_	203.08	HMDB0000929	6305	C00078
4-Hydroxyphenyllactic acid	Phenylpropanoid acids	C_9_H_10_O_4_	181.05	HMDB0000755	9378	C03672
DL-p-Hydroxyphenyllactic acid		C_9_H_10_O_4_	181.05	HMDB0000755	9378	C03672
Chrysin	Flavonoids	C_15_H_10_O_4_	253.05	HMDB0036619	5281607	C10028
3-Phenoxybenzoic acid	Benzoic acids	C_13_H_10_O_3_	213.06	HMDB0041807	19539	-
C14-SAS (TENTATIVE)	Organ sulfonic acids	C_14_H_30_O_3_S	277.18	-	-	-
Lauryl sulfate	Sulfuric acid monoesters	C_12_H_26_O_4_S	265.15	-	-	-
Tetradecylsulfate		C_14_H_30_O_4_S	293.18	-	-	-
C12-AE1S (TENTATIVE)		C_14_H_30_O_5_S	309.18	-	-	-
C10-LAS (TENTATIVE)	Benzenesulfonic acids and derivatives	C_16_H_26_O_3_S	297.16	-	-	-
Coumatetralyl	4-hydroxycoumarins	C_19_H_16_O_3_	291.10	HMDB0250496	54678504	C16806
Benzophenone-1	Benzophenones	C_13_H_10_O_3_	213.06	-	-	-

**Table 3:** List of external metabolites from cell-free supernatant (Met-Ext-CFS ESI+), classification, chemical formulas, molecular masses, and their corresponding matches (or lack thereof) in various biochemical databases, including HMDB (Human Metabolome Database), PubChem, and KEGG.

Compound	Class	Formula	Mass	HMDB	PubChem	KEGG
Tetramethylscutellarein	Flavonoids	C_19_H_18_O_6_	365.10	HMDB0030575	96118	C14472
L-Tryptophan	Amino acids	C_11_H_12_N_2_O_2_	205.10	HMDB0000929	6305	C00078
Quinidine	Alkaloids	C_20_H_24_N_2_O_2_	325.19	HMDB0015044	441074	C06527
Loperamide	Benzene and substituted derivatives	C_29_H_33_ClN_2_O_2_	477.23	HMDB0004999	3955	C07080
val-leu-pro-val-pro-gln	Oligopeptides	C_31_H_53_N_7_O_8_	244.13	-	-	-
Lincomycin	Proline and derivatives	C_18_H_34_N_2_O_6_S	407.22	HMDB0015564	656509	C06812
NCGC00385789-01_C26H42O9_5,6,16-Trihydroxygrayanotox-10-en-3-yl hexopyranoside	O-glycosyl compounds	C_26_H_42_O_9_	261.13	-	-	-
(7S,9E,11S,12R,13S,14S,15S,16R,17S,18S,19E,21Z)-2,15,17-trihydroxy-11-methoxy-3,7,12,14,16,18,22-heptamethyl-26-(octylamino)-6,23,27,29-tetraoxo-8,30-dioxa-24-azatetracyclo [23.3.1.1^4,7^.0^5,28^]triaconta-1,3,5(28),9,19,21,25-heptaen-13-yl acetate	Macrolactams	C_45_H_62_N_2_O_12_	845.42	-	-	-

**Table 4:** List of external metabolites from cell-free supernatant (Met-Ext-CFS ESI−), classification, chemical formulas, molecular masses, and their corresponding matches (or lack thereof) in various biochemical databases, including HMDB (Human Metabolome Database), PubChem, and KEGG.

Compound	Class	Formula	Mass	HMDB	PubChem	KEGG
L-Phenylalanine	Amino acids	C_9_H_11_NO_2_	164.08	HMDB0000159	6140	C00079
Tryptophan		C_11_H_12_N_2_O_2_	203.09	HMDB0000929	6305	C00078
L-(-)-Malic acid	Organic acids	C_4_H_6_O_5_	133.02	HMDB0000156	222656	C00149
DL-4-Hydroxyphenyllactic acid		C_9_H_10_O_4_	181.06	-	-	-
Pravastatin		C_23_H_36_O_7_	423.24	HMDB0005022	54687	C01844
Glimepiride	Benzenesulfonamides	C_24_H_34_N_4_O_5_S	489.22	HMDB0014367	3476	C07669
C10-LAS (TENTATIVE)	Benzenesulfonic acids and derivatives	C_16_H_26_O_3_S	297.16	-	-	-
Lauryl sulfate	Monoesters	C_12_H_26_O_4_S	265.16	-	-	-
Lithocholic acid	Monohydroxy bile acids, alcohols, and derivatives	C_24_H_40_O_3_	375.29	HMDB0000761	9903	C03990
Palmitic acid	Long-chain fatty acids	C_16_H_32_O_2_	255.24	-	-	-
Chrysin	Flavonoids	C_15_H_10_O_4_	253.06	HMDB0036619	5281607	C10028
Hydroquinidine	Alkaloids	C_20_H_26_N_2_O_2_	325.20	-	-	-

## Data Availability

The original contributions presented in this study are included in the article/Supplementary Materials. Further inquiries can be directed to the corresponding author.

## Supplementary Materials

The following supporting information can be downloaded at: https://www.mdpi.com/article/10.3390/antibiotics14020123/s1, Figure S1: Deconvolution vs. reference spectrum comparison of four compounds detected by LC-MS analysis in UTNGt2 Met-Int (ESI+) sample. Figure S2: Deconvolution vs. reference spectrum comparison of four compounds detected by LC-MS analysis in UTNGt2 Met-Ext-CFS (ESI+) sample; Table S1: Details of intracellular metabolites (Met-Int ESI+) in UTNGt2 sample; Table S2: Details of intracellular metabolites (Met-Int ESI−) in UTNGt2 sample; Table S3: Details of extracellular metabolites (Met-Ext-CFS ESI+) in UTNGt2 sample; Table S4: Details of extracellular metabolites (Met-Ext-CFS ESI−) in UTNGt2 sample; Table S5: Predicted RIPPs based on metabolite chemical structure similarity of intracellular (Esi+) compounds; Table S6: Predicted RIPPs based on metabolite chemical structure similarity of extracellular (ESI+) compounds.
