# Supplementary material for: Integrating Metabolomics and Genomics to Uncover Antimicrobial Compounds in Lactiplantibacillus plantarum UTNGt2, a Cacao-Originating Probiotic from Ecuador
Source: Antibiotics (Basel). 2025 Jan 24;14(2):123. doi: 10.3390/antibiotics14020123 (PMC11851819; doi:10.3390/antibiotics14020123)

Figure S1. Deconvolution vs. reference spectrum comparison of four compounds detected in by LC-MS analysis in UTNGt2 Met-Int (ESI+) sample. Y-Axis (Relative Abundance): Indicates the relative intensity of each ion detected. Higher peaks represent ions with greater abundance; X-Axis (m/z): Represents the mass-to-charge ratio (m/z) of ions detected. Peaks in the blue spectrum that have no corresponding red peak (or vice versa) indicate ions present in one but not the other.

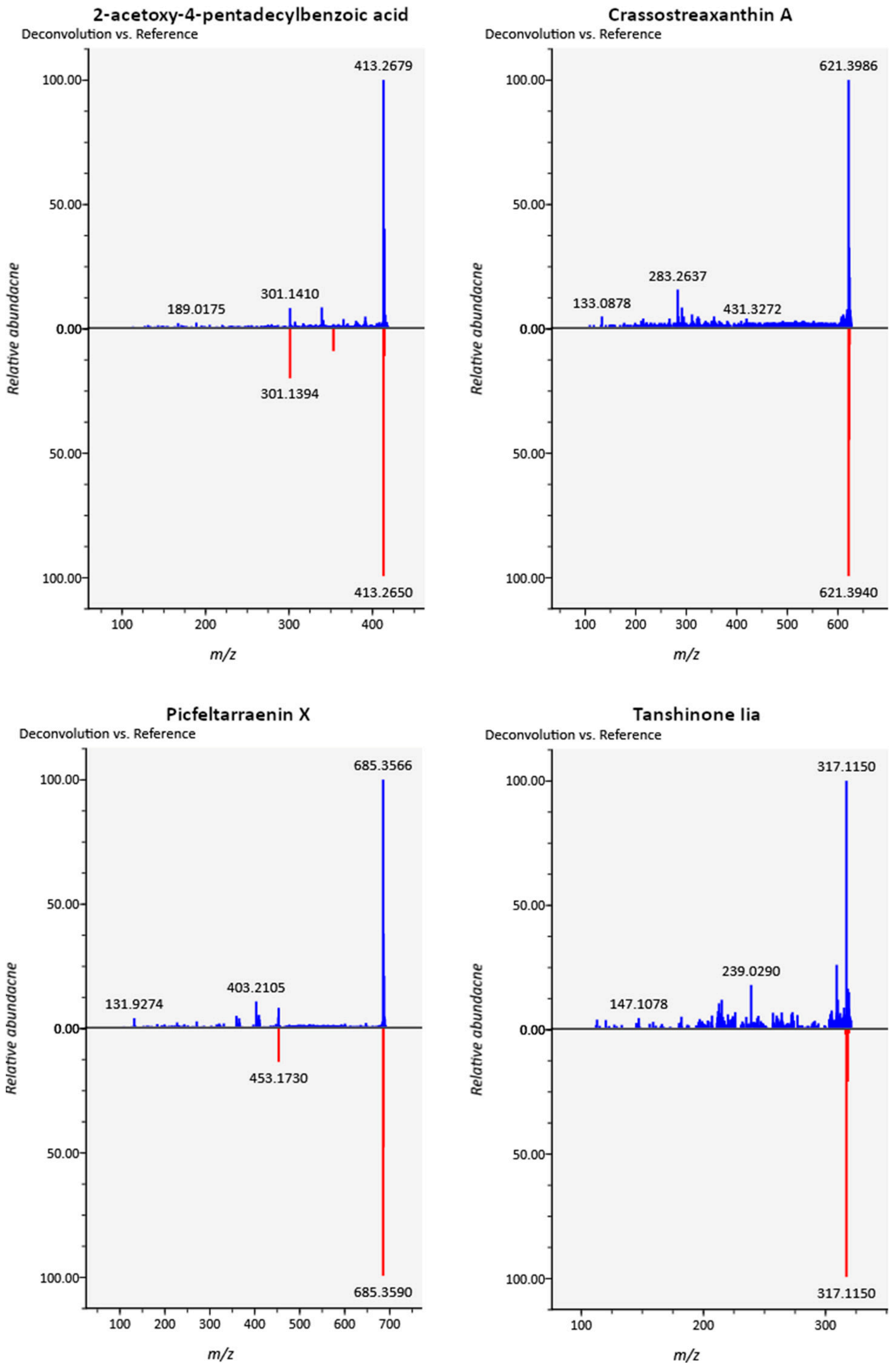

Supplement: Supplementary file 1 [file antibiotics-14-00123-s001.zip › Figure S1.pdf]
